# Improvement of cold tolerance in maize (*Zea mays* L.) using *Agrobacterium*-mediated transformation of *ZmSAMDC* gene

**DOI:** 10.1080/21645698.2022.2097831

**Published:** 2022-07-12

**Authors:** Peng Jiao, Shiyou Jin, Nannan Chen, Chunlai Wang, Siyan Liu, Jing Qu, Shuyan Guan, Yiyong Ma

**Affiliations:** a College of Life Sciences, Jilin Agricultural University, Changchun, Jilin, China; b Joint International Research Laboratory of Modern Agricultural Technology, Ministry of Education, Jilin Agricultural University, Changchun, Jilin, China; c College of Agronomy, Jilin Agricultural University, Changchun, Jilin, China

**Keywords:** Cold tolerance, Maize, *SAMDC*

## Abstract

Maize (*Zea mays* L.) is a food crop sensitive to low temperatures. As one of the abiotic stress hazards, low temperatures seriously affect the yield of maize. However, the genetic basis of low-temperature adaptation in maize is still poorly understood. In this study, maize S-adenosylmethionine decarboxylase *(SAMDC*) was localized to the nucleus. We used *Agrobacterium*-mediated transformation technology to introduce the *SAMDC* gene into an excellent maize inbred line variety GSH9901 and produced a cold-tolerant transgenic maize line. After three years of single-field experiments, the contents of polyamines (PAs), proline (Pro), malondialdehyde (MDA), antioxidant enzymes and ascorbate peroxidases (APXs) in the leaves of the transgenic maize plants overexpressing the *SAMDC* gene significantly increased, and the expression of elevated *CBF* and cold-responsive genes effectively increased. The agronomic traits of the maize overexpressing the *SAMDC* gene changed, and the yield traits significantly improved. However, no significant changes were found in plant height, ear length, and shaft thickness. Therefore, *SAMDC* enzymes can effectively improve the cold tolerance of maize.

## Introduction

Maize (*Zea mays* L.) is an annual herbaceous plant and the second-largest food crop in the world.^1^ Chilling damage has become a major adverse factor for the growth and development of maize, seriously affecting its yield.^2^ The differences in physiological and biochemical indicators and agronomic traits of plants are studied using biotechnological methods, such as genetic modification under cold stress, to provide an important theoretical basis for cultivating new cold-tolerant maize varieties.

S-adenosylmethionine decarboxylase (*SAMDC*) is one of the key enzymes in the polyamine (PA) biosynthesis pathway. It can catalyze the reaction in which S-adenosylmethionine (SAM) provides the aminopropyl group required for the synthesis reaction after decarboxylation and effectively promote the conversion of putrescine into spermidine and spermine.^3–5^ Meng et al.^6^ (2020) isolated the full-length cDNA of *SAMDC* (*AhSAMDC*) from peanuts (*Arachis hypogaea* L.). The *AhSAMDC* can effectively increase the PA content and reduce membrane damage to enhance plant resistance to salt stress. Liu et al.^7^ (2018) found that the expression of the *CmSAMDC* gene in melon was induced by powdery mildew and might be involved in the response related to powdery mildew resistance. Luo et al.^8^ (2017) proposed that the overexpression of the *SAMDC* gene could improve the cold tolerance of *Fructus edulis* by participating in the signal transductions of H_2_O_2_ and NO. Ifigeneia et al.^9^ (2016) illustrated that the overexpression of the *SAMDC* gene under salt stress could increase biomass and change developmental characteristics, such as increasing tobacco height and leaf number. Osama et al.^10^ (2010) demonstrated that the overexpression of the *SAMDC* gene could increase the level and ability of PA accumulation in cotton. Chen et al.^11^ (2018) found that cholesterol could induce the expression of the *SAMDC* gene and promote the synthesis of Spd and Spm, leading to the dwarf and drought tolerance of herbaceous plants. The *SAMDC* gene has been cloned from many plants, such as Arabidopsis, rice, and wheat.^12–14^ However, the study on the *SAMDC* gene in maize has not been reported yet.

In this study, to cultivate cold-tolerant maize lines, *Agrobacterium* was used to transform callus, and the *SAMDC* gene was overexpressed into the excellent maize inbred line GSH9901. Under cold stress, the *SAMDC* gene was overexpressed to improve the PA content and proteolytic content in the leaves. The significant increase in the contents of acid, malondialdehyde (MDA), antioxidant enzymes, and yield proves that the *SAMDC* gene can effectively improve the cold tolerance of maize.

## Materials and Methods

### Plant Materials and Cold Treatment

The seeds at the germination stage were cold treated at 4°C for 0, 2, 4, and 6 d. The WT plants and transgenic maize plants (C3) were grown in soil in a growth chamber at 25°C/75% humidity with a 16-h light/8-h dark cycle. The plants at the trifoliate stage were cold treated at 4°C for 0, 12, and 24 h. The experiment was performed in three biological repeats.

### Subcellular Localization Assay of ZmSAMDC Gene

To study the subcellular localization properties of the *ZmSAMDC* gene, we modified the pCAMBIA1302 vector to construct a fusion expression vector pCAMBIA1302-ZmSAMDC-GFP for the *ZmSAMDC* gene and the green fluorescent protein reporter gene *GFP*. The maize ubiquitin promoter was used in this vector to regulate gene expression. A control vector pCAMBIA1302 was also constructed. The recombinant plasmid pCAMBIA1302-ZmSAMDC-GFP and control plasmid pCAMBIA1302-GFP were transformed into tobacco epidermal cells by *Agrobacterium*-mediated transformation method. The infected tobacco leaf cells within 24 h were observed with an LSM710 microscope.

### Transformation and Molecular Characterization

In order to obtain transgenic maize overexpressing the *SAMDC* gene, primer pairs containing *BstE* II (5’-ACTCTTGACCATGGTAGATCTTCCCTCCATCTCCAGCATTG-3’) and *Bgl* II (5’-GGGGAAATTCGAGCTGGTCACCAACCACGAAATTGCGACGAT-3’) restriction enzyme sites were used, and the open reading frame of the *ZmSAMDC* gene was amplified. The amplified product was inserted into pCAMBIA3301 vector to replace the GUS-encoding gene *gusA*. The recombinant plasmid pCAMBIA3301-ZmSAMDC-bar was introduced into maize “GSH9901” using the *Agrobacterium*-mediated transformation method in the early stage of our research group.^15^

The T_3_ generation plants were identified using PCR by selecting bar genes with the primer pair 5′-TCAAATCTCGGTGACGGGC-3′ and 5′-ATGAGCCCAGAACGACGCC-3′ (552 bp). The protein expression of the *ZmSAMDC* gene was tested through western blot analysis in T_3_ generation plants. The protein from young leaves was fractionated by sodium dodecyl sulfate polyacrylamide gel electrophoresis (SDS-PAGE) using a Mighty Small II electrophoresis system (Hoefer Scientific Instruments, San Francisco, CA, USA).^16^ The slab gel was composed of a 12% (w/v) separation gel and a 5% (w/v) concentration gel. The leaf protein was separated and then blotted onto the PVDF membrane using the wet transfer technique. The primary antibody was added to the protein. In addition, the protein was incubated with the secondary antibody. Then, the proteins were stained in the dark with Diaminobenzidine (DAB) horseradish peroxidase (POD) coloring solution until obvious bands appeared. Finally, the sample was washed with PBST to stop the reaction, drained, and saved with pictures.

### Detection of Physiological and Biochemical Indicators

The leaves of the three transgenic lines and WT plants were sampled at the trefoil stage. High-performance liquid chromatography (HPLC) was used to determine the PA content of the plant leaves.^17^ The Pro content of the maize leaves was analyzed using the acid ninhydrin method.^18^ The thiobarbituric acid method was used to analyze the MDA content of the maize leaves.^19^ The dry weight and fresh weight analysis techniques were applied to determine the relative water content of the plant leaves.^20^ The POD content of the maize leaves was analyzed using the guaiacol method.^21^ The nitrogen blue tetrazolium method was used to analyze the superoxide dismutase (SOD) content of the maize leaves.^22^ The catalase (CAT) content of the maize leaves was analyzed using the UV absorption method.^23^ The ascorbate peroxidases (APX) activity was determined with an absorbance photometer at 290 nm (absorption coefficient 2.8 mM^−1^ cm^−1^).^24^ The experiment was repeated three times, and the average values of various physiological indicators were calculated.

### Expression Analysis of cold-responsive Genes Regulated by ZmSAMDC Gene

The cold signal pathway genes, *CBF1/2/3, RD29A, COR15A*, and *COR47*, were detected using RT-qPCR. The WT plants were used as controls, and *ACTIN2* was used as an internal reference gene. Each sample was analyzed based on three technical replicates. Each 20 µL of mixture sample was used for the following PCR cycles: 95°C for 10s and 55°C for 55s. The 2^−ΔΔCt^ method was used to calculate the relative expression changes among samples.^25^ The details regarding the primers used for this assay are listed in Supplementary Table S1.

### Field Trial Methods

The T_3_ transgenic lines overexpressing the *ZmSAMDC* gene, C3-1, C3-3 and C3-6, and “GS9901” maize (WT) were planted under natural conditions in Jilin Agricultural University genetically modified crop test base (43°47′56″N, 125°24′2″E), Nanguan District, Changchun City, Jilin Province in 2020. A completely randomized block design with three replicates was adopted for the experiment. The plants were planted in rows of 5 m long, 1 m apart, and 25 cm apart. A total of 4 plants (WT, C3-1, C3-3 and C3-6) were randomly selected during the growth period to analyze the agronomic traits after maturity (the control plant and each transformation event were repeated three times).

### Statistical Analysis

The statistical data analyses were performed using the IBM SPSS Statistics 19. One-way ANOVA was used to confirm the variability of results between treatments, respectively. Non-significant (ns), P < .05 (*) and P < .01 (**).

## Results

### Identification of Subcellular Localization of ZmSAMDC Gene in Tobacco

**Figure 1.**
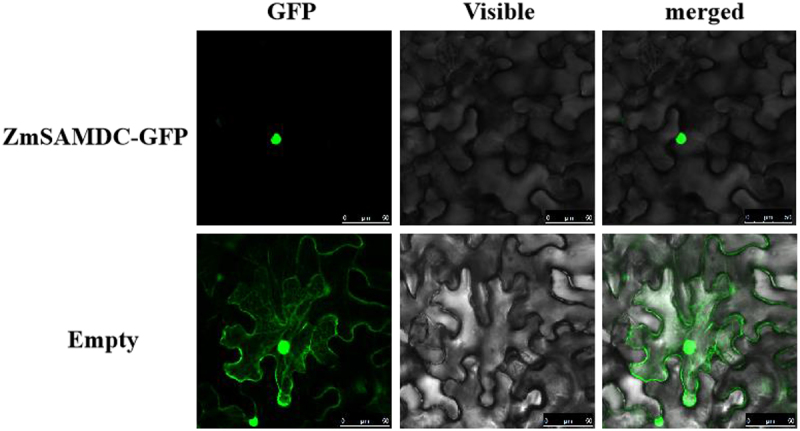
Subcellular localization analysis of the *ZmSAMDC* gene in tobacco cells. The scale bar represent 50 μm.

To study the subcellular localization of the *ZmSAMDC* gene, the target gene was cloned into the transient expression vector pCAMBIA1302-GFP using the Gateway recombination technology and then transformed into onion epidermal cells by *Agrobacterium*. The results of confocal microscopy (Olympus, Japan) showed that the *ZmSAMDC* gene transcribed proteins in the nucleus. (Fig. 1).

### The Transgenic Maize Overexpressing ZmSAMDC Gene

**Figure 2.**
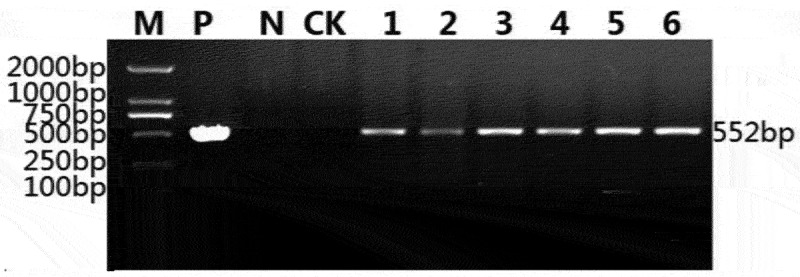
Identification of transgenic maizes overexpressing *ZmSAMDC* by PCR analysis(*bar* gene).M: DL2000 ladder, P: positive control (plasmid as template), N: negative control (H_2_O as template), CK: negative control (“GSH9901” DNA as template), 1–6: transgenic plants.

**Figure 3.**
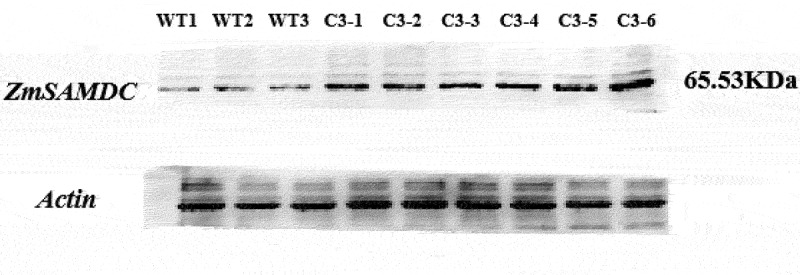
Western blot analyses of transgenic maizes overexpressing *ZmSAMDC*. WT1-WT3: negative control. C3-1~ C3-6: transgenic plants.

The PCR analysis of T_3_ generation plants using specific primers of the selection marker gene bar showed that six independent transgenic lines were obtained (Fig. 2). The Western Blot results showed that, compared with the protein expression content of the control group, that of the transgenic lines significantly increased; all of the six lines could successfully express the 65.53 kDa protein (Fig. 3). Transgenic lines, C3-1, C3-3, and C3-6, were selected to analyze the physiological and biochemical indicators and yield traits.

### Analysis of PA Content in Plants Overexpressing the ZmSAMDC Gene

**Figure 4.**
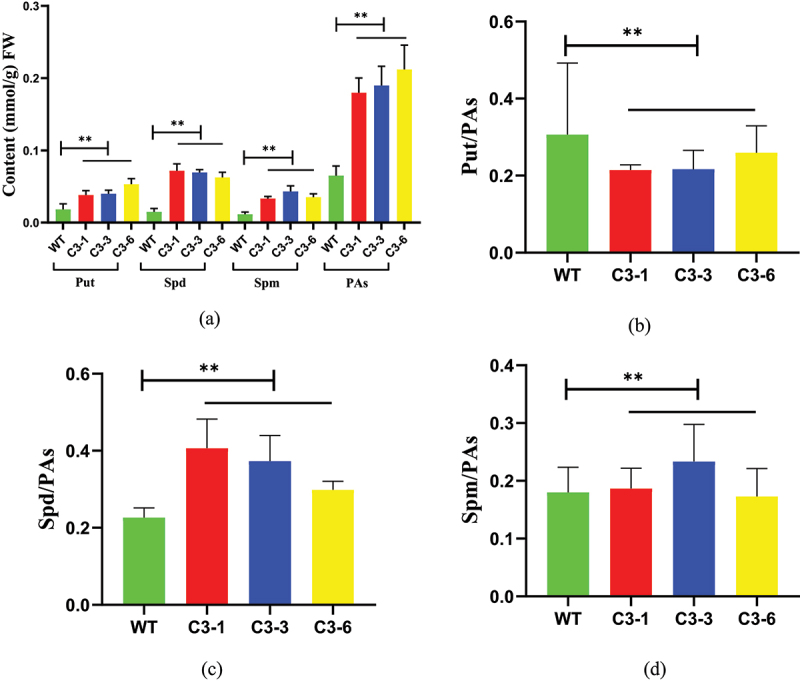
Determination of the average content of three polyamines (a) in transgenic strain C3 and The relative proportions of Put (b), Spd (c), and Spm (d) in the leaves of transgenic lines. Data were expressed as the mean of triplicate values and error represented the SD. P < .05 (*) and P < .01 (**).

As low-molecular-weight aliphatic nitrogenous bases with strong biological activity, PAs can bind to the phospholipids of cell membranes under cold stress to prevent intracellular solutes from exuding and improve the cold tolerance of plants. The average content of the three PAs of the transgenic line C3-1,C3-3 and C3-6 were higher than that of the control group (Fig. 4(a)). The relative proportions of Put, Spd, and Spm in the leaves of transgenic lines changed. The proportion of Spd in the plants overexpressing the *ZmSAMDC* gene significantly increased (Fig. 4(b-c)), while the proportion of Put significantly decreased.

### Overexpression of ZmSAMDC Gene Enhancing Cold Tolerance of Maize

**Figure 5.**
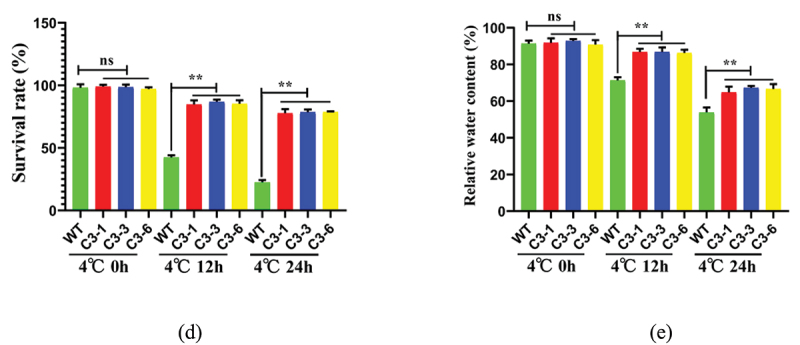
Continued.

To investigate the role of the *ZmSAMDC* gene in cold tolerance, we further analyzed the three transgenic lines overexpressing the *ZmSAMDC* gene, C3-1, C3-3 and C3-6. The RT-qPCR analysis of the three transgenic lines showed a high expression of the *ZmSAMDC* gene in maize (Fig. 5(a)). Under normal conditions, the morphological difference between transgenic lines and wild-type (WT) plants was not statistically significant. However, the germination ability of transgenic seeds was significantly stronger than that of the control group at 4°C low temperature stress for 2, 4 and 6 d (Fig. 5(b)); The damage degree of the transgenic seedling-stage lines was significantly lower than that of the control group at 4°C low temperature stress for 12, 24 and 48 h (Fig. 5(c)), and the survival rate and relative water content of the transgenic lines were significantly higher than those of the WT plants (Fig. 5(d,e)). These results indicated that the overexpression of the *ZmSAMDC* gene improved the cold tolerance of the transgenic maize.
**Figure 5.**
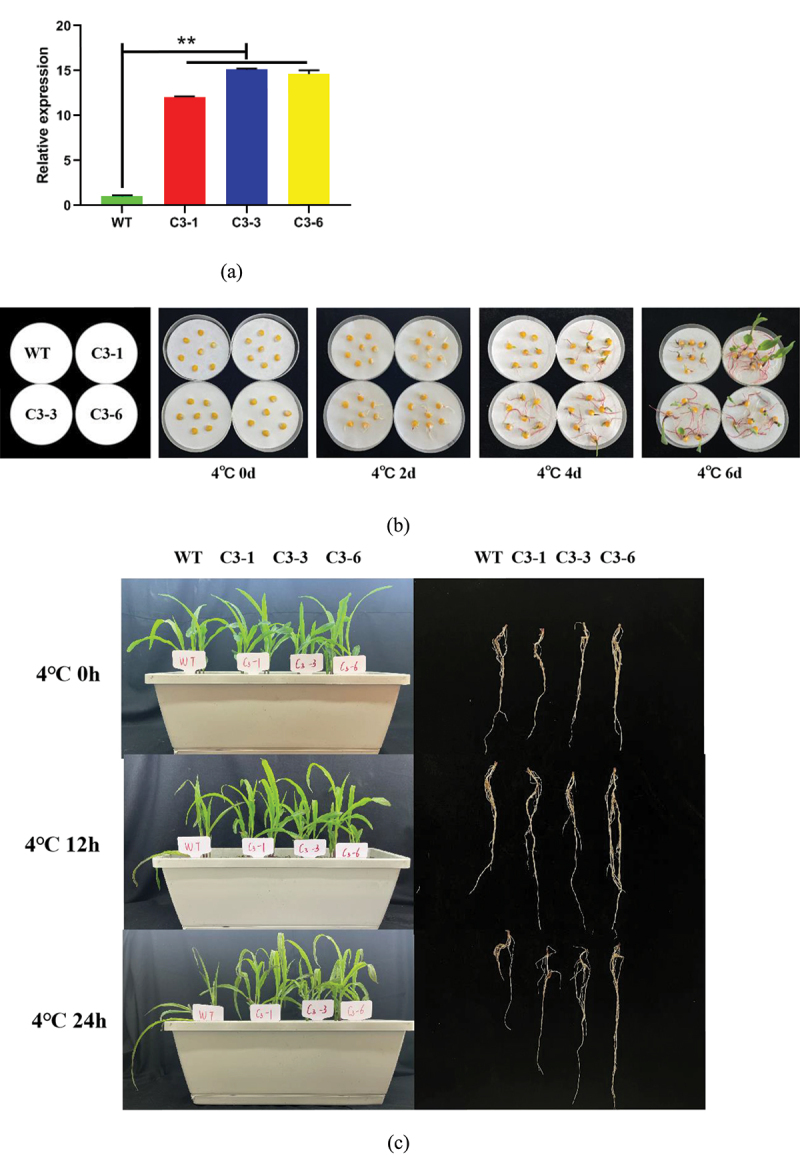
Overexpression of *ZmSAMDC* enhances the cold tolerance of maize.**a** Analysis of *ZmSAMDC* expression in positive transgenic maize. The expression level was normalized to that of Maize *ZmACTIN1*.**b** Germination phenotypes of transgenic lines and wild-type plants under non-treatment and 4°C treatment for 0,2,4 and 6 days. Analysis of wilting degree (c),survival rate (d) and relative water content (e) of transgenic lines and wild-type plants under 4°C for 0, 12 and 24 h after the three-leaf period. Data were expressed as the mean of triplicate values and error represented the SD.Non-significant (ns),P < .05 (*) and P < .01 (**).

### Overexpression of ZmSAMDC Gene under Cold Stress Significantly Increasing Leaf Proline (Pro) Content and MDA Content

**Figure 6.**
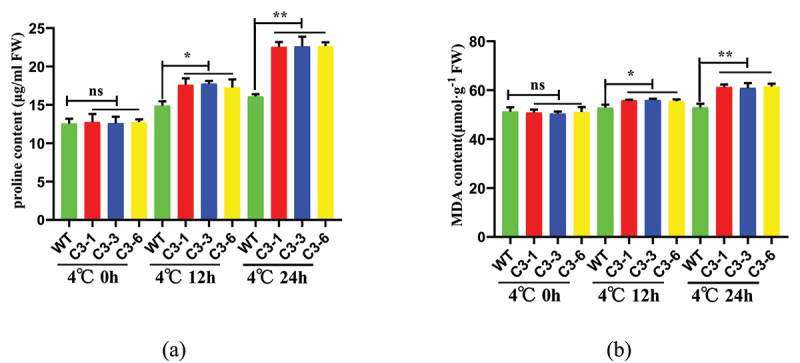
Overexpression of *ZmSAMDC* enhanced Leaf proline content (a) and MDA content (b) under 4°C treatment for 0,12 and 24 h. Data were expressed as the mean of triplicate values and error represented the SD.Non-significant (ns),P < .05 (*) and P < .01 (**).

Under cold stress, the Pro content of plants increases, and the varieties with strong cold tolerance tend to accumulate more Pro. The Pro content of the transgenic lines (C3-1, C3-3, and C3-6) showed an upward trend with the increase in the treatment time of low temperature (4°C) (Fig. 6(a)). We found that the Pro content changed the most significantly when the lines were treated at 4°C for 24 h. The average Pro content of the transgenic lines was 6.1 μg/ml, higher than that of the control group. The results indicated that the overexpression of the *ZmSAMDC* gene changed the protein composition of transgenic maize leaves, resulting in a large accumulation of Pro in plant cells.

Under cold stress, the MDA content which reflects the stress resistance of plants increased with the increase in the active oxygen content of plant leaves. With the increase in the treatment time of low temperature (4°C), the MDA content of the transgenic lines showed an upward trend (Fig. 6(b)). These results indicated that the transgenic lines accumulated relatively few reactive oxygen species (ROS) under cold stress.

### Overexpression of ZmSAMDC Gene under Cold Stress Enhancing Cold Tolerance of Plants by Increasing Leaf Antioxidant Enzymes

**Figure 7.**
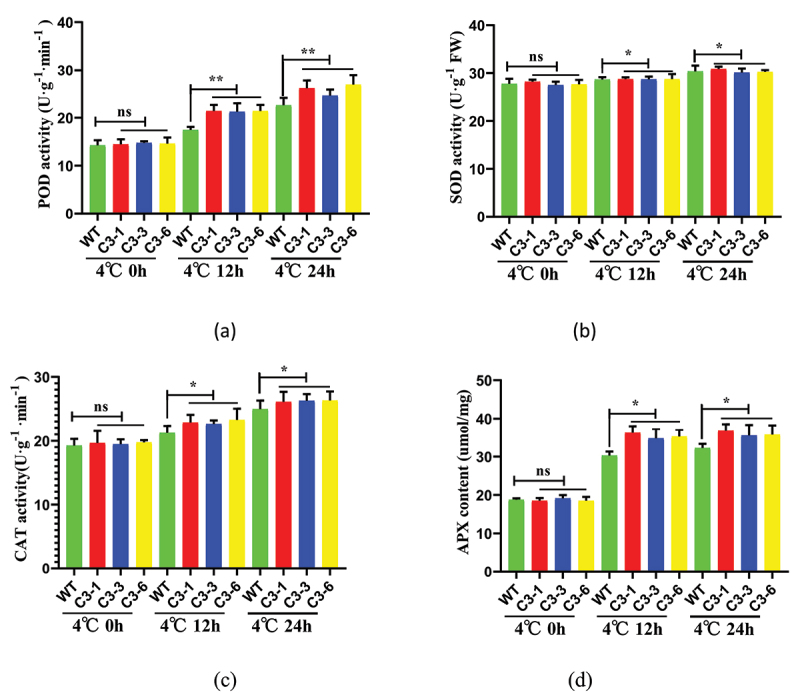
Overexpression of *ZmSAMDC* reduced reactive oxygen species (ROS) accumulation by increasing antioxidant enzyme activity under 4°C treatment for 0,12 and 24 h. (a). Analysis of peroxidase (POD) activity in leaves. (b). Analysis of superoxide dismutase (SOD) activity in leaves. (c). Analysis of catalase (CAT) activity in leaves. (d). Analysis of ascorbate peroxidase (APX) activity in leaves. Data were expressed as the mean of triplicate values and error represented the SD.Non-significant (ns),P < .05 (*) and P < .01 (**).

The metabolic system of a plant changes significantly in a cold environment. The amount of oxygen absorbed by the plant reduces, and a large amount of harmful active oxygen is accumulated, damaging the plant. The level of antioxidant enzyme activity can measure the resistance of the plant. With the increase in the treatment time of low temperature (4°C), the contents of POD, SOD, CAT, and APX of the transgenic lines increased (Fig. 7(a–d)). When the transgenic lines were treated at 4°C for 24 h, the average contents of POD, SOD, and CAT of the transgenic lines were higher than those of the control group. When the transgenic lines were treated at 4°C for 12 h, the average APX content of the transgenic lines was 5.58 μmol/mg, higher than that of the control group. Therefore, the overexpression of *ZmSAMDC* changed the oxidative stress response of the plants, improved the ability of the plants to resist oxidation, scavenge cationic free radicals and catalyze ascorbic acid (AsA), and promoted the decomposition of H_2_O_2_ in the plants.

### ZmSAMDC Gene Positively Regulating CBFs and cold-responsive (COR) Gene Expression under Cold Stress

**Figure 8.**
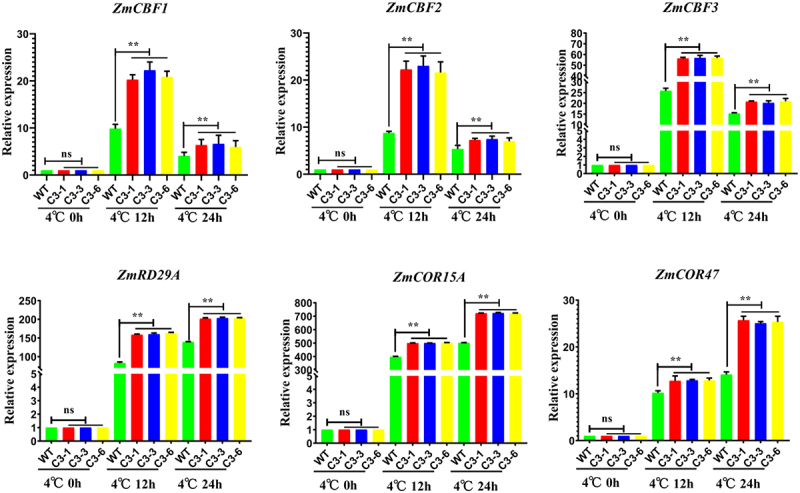
Analysis on expression patterns of cold-responsive genes in transgenic plants under 4°C treatment for 0,12 and 24 h.The expression level was normalized to that of Maize *ZmACTIN1*. Data were expressed as the mean of triplicate values and error represented the SD. Non-significant (ns),P < .05 (*) and P < .01 (**).

In order to further clarify the molecular mechanism of the lines which overexpress the *ZmSAMDC* gene responding to cold stress, we used RT-qPCR to study the expression patterns of cold-induced CBF family genes and downstream cold-responsive genes. In the transgenic lines and WT plants, the CBF family genes *CBF1, CBF2*, and *CBF3* were induced rapidly and peaked at 4°C for 12 h. However, the expression levels of these three *CBF* genes in all transgenic lines were higher than those of the WT plants. The *RD29A, COR15A*, and *COR47* genes are the downstream target genes of *CBFs*. These *COR* genes were gradually induced to be expressed under cold stress (Fig. 8). These results indicated that the overexpression of the *ZmSAMDC* gene positively regulated the expression of the *CBF* genes and downstream *COR* genes, thereby improving the cold tolerance of maize.

### Overexpression of ZmSAMDC Gene Significantly Increasing the Yield of Maize

Field experiments were conducted to observe the agronomic traits of the transgenic lines. The results are shown in Table 1. There were no significant differences in plant height, ear length, shaft thickness, and other traits between the transgenic lines and the control group, indicating that the *ZmSAMDC* gene might not affect these traits. However, the number of rows and 100-seed weight of the transgenic plants were significantly higher than those of the control group, and the bald tip length was significantly lower than that of the control group. Therefore, *SAMDC* genes may affect maize yield by regulating yield component traits.

**Table 1.** Agronomic performance of overexpressing *ZmSAMDC* lines and wild-type plants in field.

Genotype	Plant height (cm)	Ear henght (cm)	Ear diameter (cm)	The average bald tip (cm)	Kernel numbers	100-seed weight (g)
WT	121.82 ± 0.41	13.9 ± 0.67	5.66 ± 0.15	2.28 ± 0.13	30	26.32 ± 0.01
C3-1	121.72 ± 0.42	14.3 ± 0.16	5.58 ± 0.07	1.38 ± 0.02*	34*	29.32 ± 0.12**
C3-3	121.64 ± 0.4	14.3 ± 0.07	5.65 ± 0.06	1.43 ± 0.15*	35*	29.35 ± 0.25**
C3-6	121.57 ± 0.57	14.2 ± 0.13	5.45 ± 0.2	1.28 ± 0.75**	34*	29.79 ± 0.24**

## Discussion

As a key enzyme in synthesizing spermine and spermidine, the *SAMDC* gene participates in the resistance reaction of most plants. In many cases, H_2_O_2_ produced by the PA catabolism pathway is a protective measure.^26^ Diao et al.^27^ (2017) demonstrated in tomatoes that Spd and Spm induced the generation of H_2_O_2_ by increasing the activities of diamine oxidase and PA oxidase and prompting the ROS system to respond. Saha et al.^28^ (2015) also confirmed that PAs might trigger ROS synthesis or scavenge ROS, depending on the concentration of PAs in the cell. In the stress response process, PAs can stabilize the composition of molecules and maintain the integrity of cell membranes through multiple binding proteins,^29,30^ thereby eliminating ROS and reducing lipids in plants.^31^ POD can maintain membrane stability and reduce oxidative stress damage. Transgenic centipedegrass (*Eremochloa ophiuroides* [Munro] Hack.) overexpresses the *SAMDC* Gene for improved cold tolerance through the Involvement of H_2_O_2_ and NO signaling.^8^ In this study, the contents of Pro, CAT, POD, SOD, and MDA in the plants overexpressing the *SAMDC* gene were all higher than those of the negative control group, indicating that the overexpression of the *ZmSAMDC* gene influenced the reactive oxygen species and ROS system during cold stress. The results further proved that PAs affected the ROS system indirectly. The above results are basically consistent with previous studies on the overexpression of the *SAMDC* gene to improve cold, drought, and salt tolerances.^6,13,14^

Field agronomic traits are important indicators for selecting and breeding excellent new maize varieties. Bais et al.^32^ (2002) found that PAs were widely involved in fruit development and maturity, leaf senescence and stress response in plants. The *SAMDC* gene can regulate the contents of PAs, such as putrescine, spermidine and spermine, in plants to affect the biosynthesis of DNA, RNA and protein in plants, promote plant growth and development, and enhance the resistance of plants.^33^ Zhu et al.^34^ (2020) used the antisense RNA of the *SAMDC* gene to decrease the transcription level of the *SAMDC* gene rapidly. With the decrease in the contents of Spd and Spm, plants showed growth inhibition, internode shortening, stem branching, and leaf reduction. In this study, the baldness of the plants overexpressing the *SAMDC* gene was improved, and the number of rows and 100-seed weight increased, indicating that the *ZmSAMDC* gene could effectively hinder the decreased yield caused by low temperatures.

The expression of three members of the CBF family can be rapidly and transiently induced by low temperatures. Yang et al.^35^ (2019) found that the expression of *AtCBF1/2/3* was induced in *DlICE1* transgenic lines under cold stress. Chinnusamy et al.^36^ (2003) found that *AtRD29A, AtCOR15A, AtCOR47* and *AtKIN1* had multiple CRT/DRE cis-elements in the promoter region, These genes are highly induced by *AtICE1*. In this study, the overexpression of the *ZmSAMDC* gene positively regulates the expression of the *CBF* and downstream *COR* genes to improve the cold tolerance of maize.

## Conclusion

The most important finding of this study is that the overexpression of the *SAMDC* gene increases the contents of PAs, Pro, MDA, and antioxidant enzymes in the leaves to improve the yield of maize under cold stress. Therefore, engineering the SAMDC enzyme is an effective strategy to improve the cold tolerance.

## Supplementary Material

Supplemental Material
