# Supplementary material for: Improvement of cold tolerance in maize (Zea mays L.) using Agrobacterium-mediated transformation of ZmSAMDC gene
Source: GM Crops Food. 2022 Jul 12;13(1):131–41. doi: 10.1080/21645698.2022.2097831 (PMC9291676; doi:10.1080/21645698.2022.2097831)
Supplement: Supplemental Material [file KGMC_A_2097831_SM4326.doc]

Table S1.List of primers used in this study

| **Name** | **Sequence 5’-3’** |
| --- | --- |
| ZmActin1-F | ATGTTTCCTCCCATTGCCGAT |
| ZmActin1-R | CCAGTTTCGTCATACTCTCCCTTG |
| ZmSAMDC-qPCR-F | CGTGGTTGTCGTTTGGTTATTC |
| ZmSAMDC-qPCR-R | CCACCTAGAGCTGCATACTTAC |
| ZmCBF1-qPCR-F | AAGATCAAGACGAAGGATAGCATGAG |
| ZmCBF1-qPCR-R | ATCGTCTCCTCCATGTCCAG |
| ZmCBF2-qPCR-F | TGACGTGTCCTTATGGAGCTA |
| ZmCBF2-qPCR-R | CTGCACTCAAAAACATTTGCA |
| ZmCBF3-qPCR-F | GATGACGACGTATCGTTATGGA |
| ZmCBF3-qPCR-R | TACACTCGTTTCTCAGTTTTACAAAC |
| ZmRD29A-qPCR-F | AGAGGTGGTGTAACGGGTAA |
| ZmRD29A-qPCR-R | GGCTCAATGGGTTTGGTG |
| ZmCOR15A-qPCR-F | CCACCGACTCCTCTCTGCTT |
| ZmCOR15A-qPCR-R | AAGGGAGATTCCGAGATATGAAGA |
| ZmCOR47-qPCR-F | CAGTGTCGGAGAGTGTGGTG |
| ZmCOR47-qPCR-R | ACAGCTGGTGAATCCTCTGC |
